# Detailed association between the etiologies of heart failure and occurrence of sleep‐disordered breathing

**DOI:** 10.1002/clc.23875

**Published:** 2022-06-22

**Authors:** Yuri Nakagawa, Teruhiko Imamura

**Affiliations:** ^1^ Second Department of Internal Medicine University of Toyama Toyama Japan

To the Editor,

The existence, severity, and type of sleep‐disordered breathing (SDB) have a deep association with heart failure.^1^ Wang et al. investigated the association between the prevalence of SDB and each etiology of heart failure.^2^ They found that the prevalence and types of SDB varied in each heart failure etiology. Several concerns have been raised.

The authors classified the cohort into five groups according to the heart failure etiologies: ischemic, hypertensive, myocardial, valvular, and arrhythmic.^2^ However, given that these comorbidities are often overlapped, the clear classification might be challenging. For example, they defined myocardial heart failure as dilated cardiomyopathy, but other diseases, including hypertensive cardiomyopathy and cardiac amyloidosis, are frequently encountered in cardiomyopathy. Valvular diseases such as mitral regurgitation and arrhythmias such as atrial fibrillation sometimes worsen heart failure, whereas heart failures due to a variety of etiologies also have these comorbidities as secondary ones. A more detailed definition of heart failure etiology might be required.

As mentioned in their manuscript repeatedly, not only the etiologies of heart failure but also the severity of heart failure would have considerable associations with the prevalence and types of SDB.^2^ In their study, there were many differences in baseline characteristics and echocardiography parameters among five etiology groups. Their discussion was predominantly about the severity of heart failure in each etiology group and the occurrence of SDB. It might be ideal to match heart failure severity among the groups to focus on the impact of each heart failure etiology on SDB. Or it would be of great interest to discuss the association between each etiology itself, instead of heart failure severity, and occurrence of SDB.

We would like to know the clinical implication of their findings. The estimated prevalence of SDB in each heart failure etiology might help clinicians screen the occult SDB. Aggressive intervention to SDB might improve concomitant heart failure.

## CONFLICT OF INTEREST

The authors declare no conflict of interest.
